# Interferon-induced protein with tetratricopeptide repeats 5 of black fruit bat (*Pteropus alecto*) displays a broad inhibition of RNA viruses

**DOI:** 10.3389/fimmu.2024.1284056

**Published:** 2024-02-19

**Authors:** Emily Clayton, Mustafa O. Atasoy, Rania F. El Naggar, Ana Cláudia Franco, Mohammed A. Rohaim, Muhammad Munir

**Affiliations:** Division of Biomedical and Life Sciences, Faculty of Health and Medicine, Lancaster University, Lancaster, United Kingdom

**Keywords:** IFIT5, bats, innate immunity, virus, interferons

## Abstract

Bats are natural host reservoirs and have adapted a unique innate immune system that permits them to host many viruses without exhibiting symptoms. Notably, bat interferon stimulated genes (ISGs) have been shown to play antiviral roles. Interferon induced protein with tetratricopeptide repeats 5 (IFIT5) is a well-characterised ISG in humans with antiviral activities against negative-sense RNA viruses via inhibiting viral transcription. Here, we aim to investigate if *Pteropus alecto* (pa) IFIT5 (paIFIT5) possess the ability to inhibit negative-sense RNA viruses. Initially, gene syntenic and comparative structural analyses of multiple animals highlighted a high level of similarity between *Pteropus alecto* and human IFIT5 proteins. Our results showed that paIFIT5 was significantly inducible by viral and dsRNA stimulation. Transient overexpression of paIFIT5 inhibited the replication of vesicular stomatitis virus (VSV). Using minireplicon and transcription reporter assays, we demonstrated the ability of paIFIT5 specifically to inhibit H17N10 polymerase activity. Mechanistically, we noticed that the antiviral potential of paIFIT5 against negative sense RNA viruses was retributed to its interaction with 5’ppp containing RNA. Taken together, these findings highlight the genetic and functional conservation of IFIT5 among mammals.

## 1 Introduction

Interferons (IFNs) are a group of cytokines that are expressed and secreted as the first line of defence against viral invasion, making up a key part of the innate immune response (1). Upon infection, IFNs and their antiviral effectors are induced to limit the viral replication and virus-associated pathologies *via* eliciting an antiviral state in host cells (2). During the innate immune response, viruses are detected by host pathogen recognition receptors (PRRs) in host cells, which play critical roles in distinguishing self-molecules from non-self *via* the recognition of viral molecular signatures known as pathogen-associated molecular patterns (PAMPs). There are different types of PRRs present in the cell cytosol or endosomes, including; Toll-like receptors, RIG-I-like receptors and nucleotide oligomerisation (NOD)-like receptors, which all differ in their specific targets but are collectively responsible for foreign nucleic acids sensing (3). PAMPs are distinct conserved molecules that are not usually present in the host cellular RNA, such as 5’triphosphate (5’ppp) or 5’diphosphate (5’pp) groups and double-stranded RNA (dsRNA) (4). Following interaction with PAMPs, PRRs trigger several signaling cascades that stimulate the production of IFNs in infected cells generating an antiviral state in infected and neighbouring cells to limit the spread of viral pathogen in the host (5). In humans, type I and type III IFNs are considered the key antiviral IFNs, however type II IFNs have also been reported to possess an antiviral potential (6). IFNs bind to their cognate receptors and activate the JAK-STAT pathway leading to the transcription of hundreds of ISGs, which have antiviral and immune-modulatory roles. There are also a subset of ISGs that are induced in an IFN-independent manner following viral infection (7). ISGs are directly responsible for protecting host cells against viral pathogenesis by targeting certain steps of the viral life cycle such as viral entry, transcription, translation and virion assembly (8).

Interferon induced proteins with tetratricopeptide repeats (IFITs) are well-studied ISGs that are known to play essential roles in antiviral responses, nucleic acid sensing and protein translation in humans, whereby they can directly recognise the viral RNA molecular signatures (9). All IFIT proteins contain a characteristic feature of several tetratricopeptide repeats (TPRs), a motif composed of 34 amino acids in a helix-turn-helix structure to allow for protein-protein interactions (10). Under basal conditions, IFIT proteins are not expressed, however upon viral infection, IFIT genes are rapidly transcribed to high levels. Moreover, IFIT gene expression can also be induced directly via PAMP recognition such as dsRNA, a common by-product of infection with RNA viruses, which occurs independently without IFN stimulation (11). In this case, these genes are often referred to as viral stress-inducible genes (VSIG), which are often induced directly by IRF3, activated following viral infection (12). The IFIT family is comprised of four proteins in humans (IFIT1, IFIT2, IFIT3 and IFIT5) which are all located on chromosome 10q23 and induced via IFNs, viral infection or PAMP recognition (7). Human IFIT1, IFIT2 and IFIT3 possess analogous functions as interacting heterodimers or oligomers to bind directly with eukaryotic initiation factor 3 (eIF3). These IFIT genes are well characterized and known to potentiate diverse antiviral processes (13, 14). However, IFIT5 remains less well-understood, does not partake in the association of IFIT1, IFIT2 and IFIT3 and lacks any other interacting protein partner (13, 15).

IFIT5 gene is present in human cells, but absent in mice and rats (6) and is the only IFIT protein present in opossums, chickens, frogs and zebrafish (14). IFITs have been previously characterized to commonly restrict the viral replication via alteration of protein synthesis. Recent studies have shown that some IFIT proteins, including IFIT5 can act in an explicit manner via the direct binding with viral RNA that possessing a 5’ triphosphate group (5’ppp) at their 5’ terminus (14). IFIT5, in addition to IFIT1, is able to distinguish between cellular and viral mRNA via the detection of 5’ triphosphate group (5’ppp) at the 5’ terminus (13). IFIT5 is able to distinguish viral RNA from host RNA such as ribosomal RNAs (rRNAs) and transfer RNAs (tRNAs) as host RNAs tend to carry a cap structure at their 5’ termini consisting of a N7-methylguanosine linked to the first transcript nucleotide via a 5’-5’ triphosphate bridge, which is important for initiating translation (16). Methylation occurs at the 2’-0 position of the first or second base yielding cap1 or cap2 (m^7^GpppNmN or m^7^GpppNmNm, respectively), and although these are not essential for translation, human IFIT1 and IFIT5 can inhibit the translation of mRNA lacking cap1 (17). Several viruses are known to mimic these features through immune evasion strategies. However, negative-sense single-stranded RNA viruses such as Newcastle disease virus (NDV) and influenza A virus (IAV) do not possess any cap structures, IFIT5 can directly recognise their RNA as a foreign RNA and bind to the 5’ppp group (18). These PAMPs are recognised by PRRs on host cells, which initiate the innate immune responses to limit the viral replication (2). Recent research has suggested that alongside its direct interaction with 5’ppp, human IFIT5 has further antiviral capacity in synergising the interaction of interferon regulatory factor 3 (IRF3) and Nuclear Factor kappa-light-chain-enhancer of activated B cells (NF-κB) to mediate the gene expression (19).

Bats, belonging to the Chiroptera order, exhibit remarkable diversity with over 1,400 distinct species, making them one of the most widespread and diverse vertebrates globally (20). These creatures are recognized as significant hosts of numerous viruses, several of which pose a threat to humans, including coronaviruses, lyssaviruses, filoviruses, and henipaviruses (21). Through zoonotic spillover events, bats can transmit these viruses to humans and other mammals. Bats are considered to be potentially the most vital viral reservoir among mammals due to their capacity for zoonotic transmission and their diverse range of viral species (21, 22). Interestingly, except for lyssaviruses, tacaribe virus and lloviu virus, bats have the ability to host various viruses without manifesting any noticeable disease symptoms (23). This capacity is attributed to several distinct immune mechanisms possessed by bats. These mechanisms include the continuous activation of interferon (IFN) and the ongoing activity of interferon-stimulated genes (ISGs). These mechanisms enable bats to prevent disease manifestation while still accommodating viral replication and shedding. This occurs through the establishment of a unique equilibrium between the viruses and the host (24, 25).

Genomic and transcriptomic analyses in bats have identified a high degree of conservation of their immune systems with that of humans and other mammals, such as the presence of PRRs, IFNs and several ISGs (21). Despite bats sharing several immunological features with other mammals, there is a little research directed towards understanding their immune mechanisms and antiviral responses, due to the limited availability of resources required for bat immunological studies (26, 27). IFIT5 has been identified in several animal species including bats but remains poorly characterised both genetically and functionally. Due to the observed conservation of bat and human immune pathways and the antiviral capabilities of human IFIT5, we sought to uncover the genetic and functional implications of *Pteropus alecto* IFIT5 in interfering with the replication of viruses.

## 2 Materials and methods

### 2.1 Data collection and bioinformatic analyses

The ifit5 gene was amplified from NDV-stimulated *Pteropus alecto* cells and sequenced in both directions. The sequence was found to be identical to the IFIT5 available in the GenBank under (XM_006925963) and was used in this study. Additionally, for bioinformatic analysis, IFIT5 sequences were retrieved from the NCBI database in a FASTA format. Gene synteny was assessed via NCBI genomic sequence to observe neighbouring genes to IFIT5 on the forward strand. Sequence alignment was conducted using BioEdit software using the ClustalW Multiple Sequence Alignment algorithm. Phylogenetic analyses were generated in MEGA 11.0 software using the Maximum-Likelihood method with a bootstrap value of 1000. Using Sequence Demarcation Tool (SDT) software, pairwise % identity was generated, aligns every unique pair of sequences, and calculates the pairwise identity scores as 1-(M/N), where M is the number of mismatching nucleotides and N is the total number of positions along the alignment. Predicted 3D structures were obtained using the I-TASSER database, and then we used PyMOL software for domain annotation.

### 2.2 Plasmids

The open reading frames (ORFs) encoding Mx1 gene of *P.alecto* and the IFIT5 genes of both *P.alecto* and human, were fused with a FLAG-tag in the N-terminus, codon optimized, chemically synthesised and cloned into a pEF-pLINK vector. In addition, *P.alecto* IFIT5 was shuttled into a pEF-pLINK vector carrying a V5 tag in the N-terminus.

### 2.3 Cell culture, media and transfection

HEK293T (ATCC) and VeroE6 cells (Public Health England, now DHSA), were grown in Gibco Dulbecco’s modified eagle medium (DMEM) + GlutaMAX (10% (v/v) FBS) (Gibco), 1% (v/v) penicillin streptomycin (Gibco) at 37°C 5% CO_2_. PaBr cells (supplied by the University of São Paulo, Brazil) were grown in Gibco DMEM/F12+ GlutaMAX (10% (v/v) FBS), 1% (v/v) penicillin-streptomycin and 1% MEM non-essential amino acids (NEAA) (Gibco). PaBr cells were transfected with paIFIT5 at a ratio of 1:6 using Viafect transfection reagent (Promega). HEK293T and VeroE6 cells were transfected with huIFIT5 or paIFIT5 using Lipofectamine 2000 transfection reagent (Thermo-Scientific) at a 1:3 ratio.

### 2.4 Interferon-β production

*Pteropus alecto* interferon-β (paIFNβ)-encoding plasmid was sourced from GeneArt, Thermo-Fisher and used for the *in vitro* production of paIFNβ. Briefly, HEK293T cells were grown in 6-well plates with 80% confluency before being transfected with 3μg of IFNβ at a 1:3 ratio using Lipofectamine 2000 following the manufacturer’s recommendations. At 24, 48 hours post-infection, supernatants were collected, pooled together, centrifuged at 1500rpm for 5 minutes for clarification from any cell debris, then aliquoted into 1ml tubes and stored at -80°C to be used in subsequent IFN-stimulation studies.

### 2.5 RT-qPCR of paIFIT5

The PaBr cells were treated with either paIFN-β, polyI:C (Thermo-Scientific) or infected with NDV at MOI of 1.0. For stimulation, 200 units of paIFNβ was used and calculated as previously described using a VSV-based bioassay (28) compared to untreated cells as a control. Total RNA was extracted using TRIzol reagent (Thermo Fisher, USA) according to manufacturer’s instructions. Quantity and quality of the RNA was assessed via Nanodrop spectrophotometer (Thermo-Scientific). A total of 200ng of RNA was used for RT-qPCR using SuperScript® III Platinum® SYBR® Green One-Step qRT-PCR Kit (Thermo Fisher, USA). The reaction was carried out in an ABI 7500 light cycler (Thermo Fisher, USA) using the manufacturer’s instructions. The paIFIT5 targeting primers (qPA-IFIT5F- 5’-GGATCCCGCTCCTGAGAAAG-3’ and qPA-IFIT5R- 5’-GTCTGAGTGTTCACGCTGGA-3’) and housekeeping genes primers (qPA-18S-F: CGGCTACCACATCCAAGGAA, qPA-18S-R: GCTGGAATTACCGCGGCT) were designed and used considering MIQE guidelines (29). Quantification of paIFIT5 was performed using 2(-Delta Delta C(T) method (30).

### 2.6 Immunofluorescence

HEK293T cells or VeroE6 cells were grown on Nunc™ Thermanox™ coverslips (VWR, UK) in 24-well plates (Thermo Fisher, USA) and transfected as previously described in section 2.3. At 24 hours post-transfection, wells were washed with 300µl PBS and then fixed in 4% paraformaldehyde in PBS (Thermo Fisher, USA) for 1 hour on a rocker. Following washing with 300µl PBS; 500µl of 0.1% Triton-X100 (Sigma) was added for 10 minutes followed by washing with PBS and then 500µl 0.5% bovine serum albumin (BSA) (Sigma-Aldrich) was added for blocking for 1 hour. Cells were incubated with primary antibodies raised against V5 tag, or FLAG tagged at a concentration of 1:1500 for 2 hours. Subsequently, cells were incubated with corresponding secondary antibodies at a concentration of 1:3000 for 2 hours after 3X washing with PBS. Cells were washed 3X with PBS for 5 minute each time and then stained with 4’,6-di-aminidino-2-phenylindole (DAPI) (Thermo Fisher, USA) for 20 minutes (1:10000). Cell-coated coverslips were directly mounted onto glass microscope slides using Vectashield mounting medium (Vector). Slides were visualised and imaged using a Zeiss LSM880 confocal laser scanning microscope.

### 2.7 Virus infections and quantifications

VSV expressing green fluorescent protein (GFP) (VSV-GFP) for infection studies were generated as previously described (28). VeroE6 cells were infected with VSV-GFP at an MOI of 0.25. Briefly, media was aspirated from wells; virus was added to cells and incubated at 37°C, 5% CO_2_ for 1 hour with shaking every 20 minutes to ensure even distribution of virus. Infection media was removed, replaced with fresh growth media and cells were incubated for 24 hours. The supernatants were collected 24 hours post infection and used for plaque assay.

Plaque assay was conducted as previously described with slight modification. VeroE6 cells were grown in 12-well plates to reach 100% confluency, 10-fold serial dilutions of the viral supernatant were prepared in DMEM without serum. Media was removed and cells were washed with 500µl PBS, then the viral dilutions were added to the cell’s monolayer. Following virus incubation, infection media was removed, and cells were washed with 500µl PBS. 1.5ml of complete overlay media (1:1 mixture of 2X media and 2.4% (w/v) carboxymethylcellulose (CMC) solution supplemented with 1X antibiotic/antimycotic) was added to each well, and cells were incubated at 37°C, 5% CO_2_ for 3 days. Cells were fixed with 1ml/well of 4% paraformaldehyde for 1 hour and 1ml of 0.2% crystal violet was added for 1 hour for staining. The wells were washed with water to eliminate any surplus dye and then turned upside down for dryness. Viral titre (PFU/ml) was calculated as the average number of plaques per well/(dilution x infection volume).

### 2.8 H17N10 VLP system

H17N10 VLPs (Georg Kochs, Germany) were produced and measured as previously described (31) in the presence of paIFIT5. Briefly, HEK293T cells were transfected in a 12-well with the expression plasmids coding for PB2 (50ng), PB1 (50ng), PA (10ng) and NP (100ng) of H17N10. In addition, expression plasmids encoding the viral minigenome Pol-I FF-Luc (50ng),HA (100ng), neuraminidase (NA; 100ng), M1 (125ng), M2 (20ng) and nuclear export protein (NEP) (25ng) of SC35M (H7N7) were transfected as previously described (31) along with 300ng of the paIFIT5. As a control for functional VLP production, a plasmid encoding the HA was omitted. At 48hrs post-transfection, the firefly luciferase activity in the cell lysates was measured using the TECAN luminometer.

### 2.9 RVFV polymerase assay

In order to determine the influence of paIFIT5 on Rift Valley fever virus (RVFV) polymerase activity, HEK293T cells were transfected with expression plasmids, encoding paIFIT5 (250ng), RVFV L, M and N proteins (250ng each), a minigenome construct coding for the full-length RVFV with the NSs ORF replaced by *Renilla* luciferase (250ng) and firefly luciferase under the control of the constitutively active SV40 promoter (50ng). At 4 hours post-transfection; media was removed, replaced with fresh growth media and cells were incubated for 48 hours at 37°C. Cells were lysed and the luciferase activities were measured. The activity of *Renilla* luciferase was normalised to firefly luciferase and the empty vector control with RVFV L omitted was set to 100%.

### 2.10 *In vitro* transcription of 5’ biotinylated synthetic RNA

*In vitro* transcription and biotinylation were conducted as previously described by Santhakumar et al. (28). The 7SK-as plasmid, encoding for antisense 7SK RNA, was first linearized using BamHI (NEB, UK), and the purified DNA was used to generate *in vitro* transcribed RNA in the presence of bioin-16-UTP using RiboMAX™ Large Scale RNA Production System-SP6 (Promega, Cat# P1280) as reported previously (32). Briefly, 100µl were generated containing 20µl SP6 buffer, 10µl NTP-bioUTP mixtures, 5µg linearized plasmid and 10µl enzyme mix. The reaction mixture was then incubated at 37 °C for 3 hours. The reaction was treated with RNase-free DNAse (Thermo Fisher, USA) for 30 minutes at 37°C, to remove undigested DNA Following *in vitro* transcription, RNA was run on agarose gel electrophoresis to assess the RNA quality and subsequently purified using RNeasy MinElute Cleanup Kit (Qiagen). The purified *in vitro* transcribed and biotinylated ppp-RNA was then dephosphorylated using alkaline phosphatase (FastAP, Fermentas, Japan) to remove 5′ triphosphate (ppp), leaving an OH group, along with mock-treated. Finally, the biotinylated RNA samples were purified with RNAeasy MinElute Cleanup Kit and eluted in nuclease-free water for further use in RNA-protein interactions.

### 2.11 RNA protein immunoprecipitation for confirmation of IFIT5-binding RNA

To purify the IFIT5-binding RNAs, streptavidin affinity resin was incubated at 4°C for 2 hours with either 1µg PPP-RNA or 1µg OH-RNA as previously described (28). To prepare paIFIT5 or huIFIT5 protein, HEK293T cells (1×10^6^) were transfected with 5 μg V5-tagged paIFIT5 or huIFIT5 plasmid for 48 hours and lysed with TAP buffer in the presence of protease and RNAse inhibitors. paIFIT5 or huIFIT5 protein (2mg) lysate were incubated with the RNA-coated beads (bearing either ppp-RNA or OH-RNA) for 4 hours at 4 °C on a rotary wheel then washed 3X to remove unbound proteins. These beads were mixed with 2X loading buffer and loaded directly on SDS before probing with anti-V5 primary antibodies followed by incubation with IRDye-labelled secondary antibodies (Li-Cor Biosciences). Signals were acquired and assessed using the Li-Cor imaging system (Li-Cor Biosciences).

### 2.12 Statistical analyses

Utilising GraphPad Prism 8 software, a pairwise Student’s t-test was employed to assess the antiviral impact of paIFIT5 in comparison to the control. Mean values along with their corresponding standard errors were accurately presented in each figure. In cases where multiple comparisons were necessary for a single factor, a One-Way ANOVA was utilized.

## 3 Results

### 3.1 Genomic, structural and evolutionary characterisation of paIFIT5 locus

IFITs have been identified in several species, including most mammalian species. However, functional characterisation of IFIT genes has only been undertaken in few species (7). IFIT genes are encoded in bats genome (Ensembl Database), specifically IFIT5, but its sequence homology to IFIT5 homologues of other species had not yet been explored.

**Figure 1:**
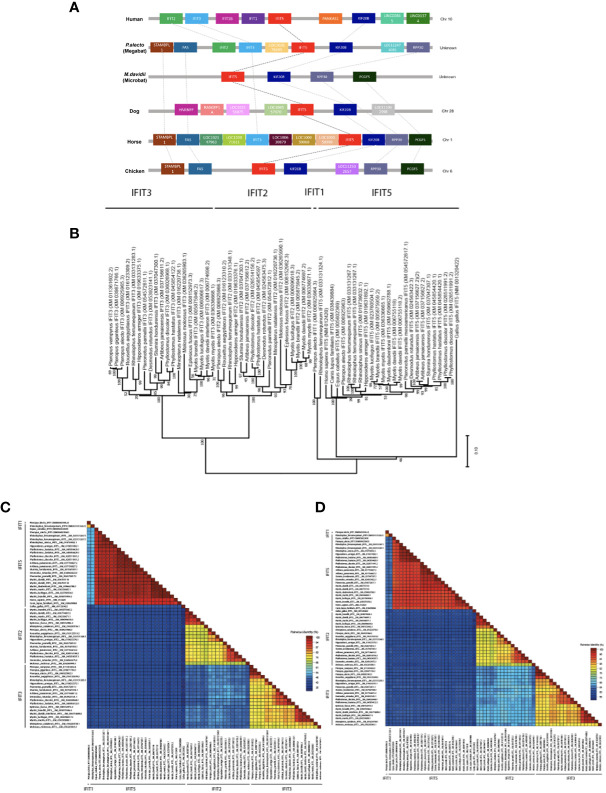
Genomic analysis, phylogenetic relationships and loci identification of IFIT5 genes in human, megabat and microbat, dog, horse and chicken. **(A)** Direct syntenic analysis of *P.alecto* IFIT5 with other species commonly known to possess IFIT5. The IFIT5 gene lies on the forward strand and is commonly flanked upstream by IFIT3 and/or FAS, and downstream by KIF20B. **(B)** Phylogenetic analysis of IFIT5 genes in different species. Bootstrap probabilities are denoted at the branch nodes. The scale bar at the bottom represents the error rate. **(C)** Pairwise % identity analysis of IFIT5 genes shows a high conservation of bat IFIT5 with other mammals. **(D)** Pairwise % identity analysis of IFIT5 proteins shows a high conservation of bat IFIT5 proteins with other mammals.

To evaluate the conservation of bats IFIT5 gene collinearity compared to other IFIT5 homologues of different species on a chromosomal level, we selected species of interest closely related to bats such as human, horse, alongside dog and chicken for a broader analysis. Distribution of IFIT5 homologues showed that they were allocated on various chromosomes in different species, as shown in **Figure 1A** (33, 34). It is worth noting that in both bat species, the chromosomal location of IFIT5 still remains unknown due to lack of genetic mapping within these species. Syntenic analyses demonstrated that IFIT5 is commonly flanked upstream by IFIT3 and/or FAS genes and downstream by KIF20B in most species studied here (**Figure 1A**). Overall, synteny remained largely conserved amongst the mammalian species, with loss of synteny in chicken due to the absence of common neighbouring genes to IFIT5. In addition, *Myotis davidii* (*M. davidii)* only displayed a limited number of genes, which is likely due to incomplete genomic annotation within these bat species. Phylogenetic analysis of *Pteropus alecto* IFIT5 (paIFIT5) and IFIT homologues (IFIT1, IFIT2, and IFIT3) along with other species of bats demonstrated that paIFIT5 made a distinct group along with mammals (**Figure 1B**). While human and dogs IFIT5 were in the same cluster, paIFIT5 was closer to horse IFIT5. The close phylogenetic relationship between bats and horses has been previously described, classifying both species within a superorder named *Pegasoferae*, encompassing *Chiroptera*, *Periossodactyla, Carnivora* and *Pholidota* (35). Therefore, the close genetic relationships observed between bat and horse IFIT5 is expected because bats are more closely related to horses than human. Further confirmation for this clustering relationship was examined via pairwise nucleotide (**Figure 1C**) and amino acid (**Figure 1D**) identity analysis between different IFIT5 sequences. Percentage identity between all mammalian species was over 96%, whilst chicken expectedly displayed a 64% identity to paIFIT5 and other mammalian species. Based on the observed clustering patterns and homology, it is apparent that paIFIT5 is highly conserved with IFIT5 genes of other mammalian species (the closest being the microbat species *M. davidii*, followed by horse, human and dog). Collectively, gene syntenic analyses, phylogeny and pairwise annotations indicate that paIFIT5 is highly genetically analogous to IFIT5 genes of other mammals (including bats).

**Figure 2:**
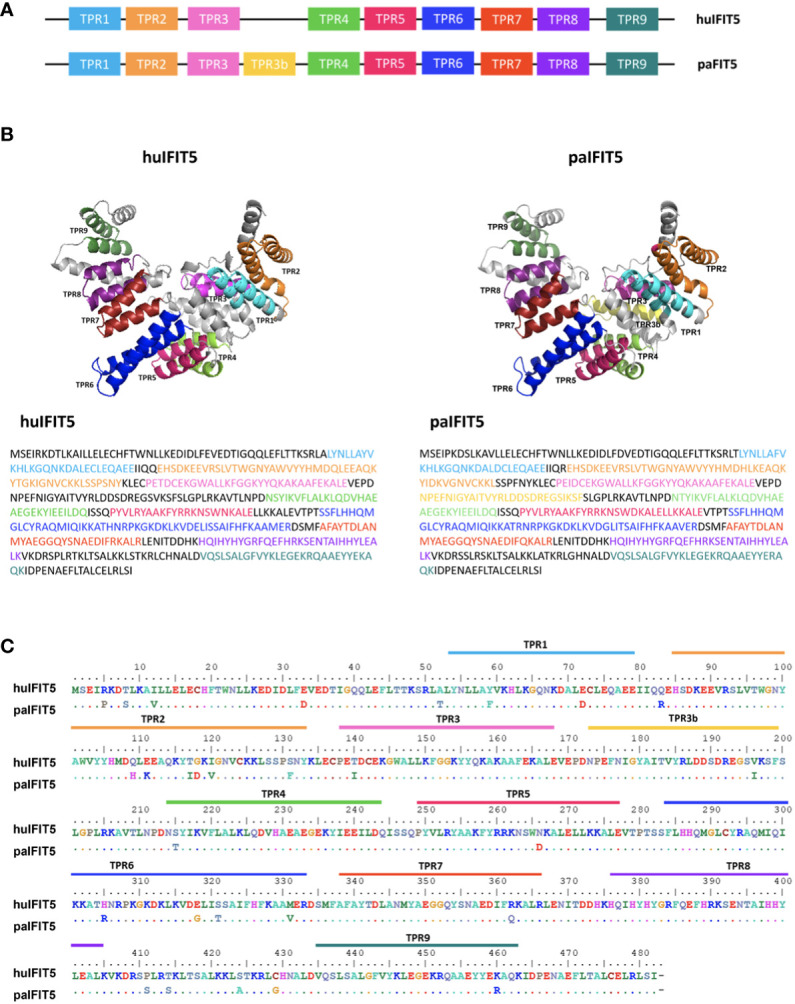
Structural overview and sequence conservation of bat (*P.alecto*) IFIT5 with human IFIT5. **(A)** TPR numbers and positions in huIFIT5 and paIFIT5. **(B)** Three-dimensional (3D) protein structure and sequence comparison between huIFIT5 and paIFIT5 displays putative homology of TPR repeats. **(C)** Sequence alignment of the entire IFIT5 protein between human and *P.alecto*, displaying homology between the two proteins and the location of TPR repeats in IFIT5 gene sequence. Amino acids in paIFIT5 sequence are represented by a dot if conserved with huIFIT5 or alternatively labelled if they differ at that position. Alignment was generated in BioEdit using the ClustalW multiple sequence alignment with a bootstrap value of 1000.

TPRs are a defining structural characteristic for all IFIT proteins, including IFIT5 (3). TPRs consist of degenerate helix-turn-helix motifs comprised of 34 amino acids, which extend throughout the length of the IFIT protein as tandem arrays and responsible for protein-protein interactions (9, 36). In this study, human IFIT5 (huIFIT5) and paIFIT5 sequences were used to predict TPRs using NCBI’s Conserved Domain Database for a direct comparison of TPR homology between the two species. The TPR number and position was highly conserved between huIFIT5 and paIFIT5, however, paIFIT5 was shown to possess an additional TPR located between TPR3 and TPR4, which was tentatively labelled as TPR3B (**Figure 2A**). Subsequently, 3D-structures were generated and annotated using Pymol software, where individual TPR sequences were labelled (**Figure 2B**). Overall, the paIFIT5 protein sequence was highly conserved compared to huIFIT5 homologue, differing only by few amino acids (**Figure 2C**). Taken together, these results highlight a high conservation between huIFIT5 and paIFIT5 along with the characterisation of their TPR repeats.

### 3.2 Subcellular distribution of IFIT5 proteins

**Figure 3:**
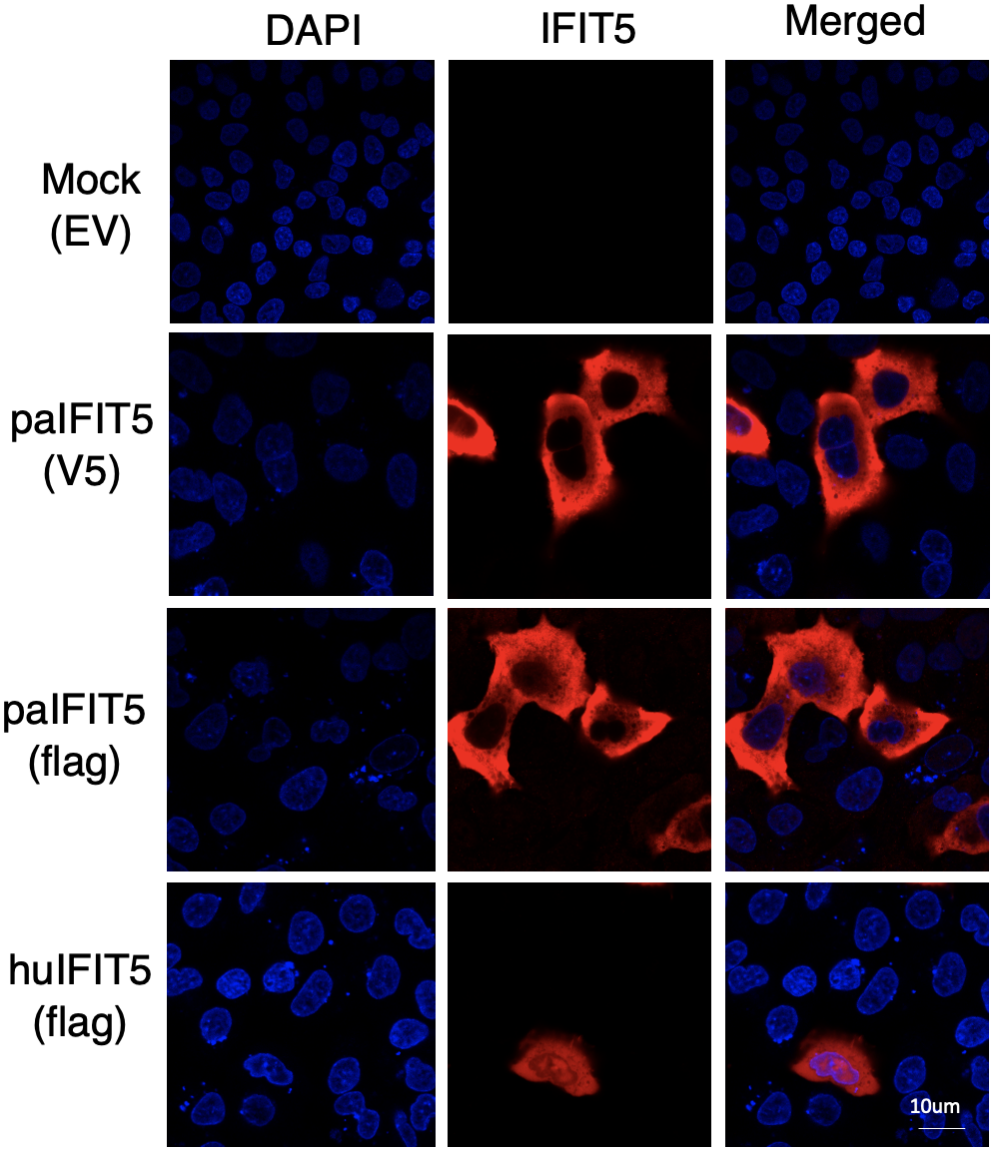
Subcellular distribution of paIFIT5 and huIFIT5 proteins expressed in VeroE6 cells. VeroE6 cells were transfected with 2µg of FLAG-tagged paIFIT5 or huIFIT5 or V5-tagged paIFIT5 for 24 hours before fixation, staining for nuclei (DAPI) and IFIT5 (tag staining). Scale bar represents 10um.

There is currently limited information available on the cellular localisation of IFIT5 proteins. Preliminary analysis predicts an intracellular localisation of huIFIT5 along with its potential localisation on the plasma membrane of A-431 and SK-MEL-30 cells (37). Bat IFIT5 (paIFIT5) subcellular localisation also remains entirely unspecified. Therefore, we aimed to identify the subcellular locations of paIFIT5 compared to huIFIT5 in mammalian (VeroE6) cells (**Figure 3**) to allow for a direct comparison between the two proteins. After transfection, both paIFIT5 and huIFIT5 were fixed and stained using primary antibodies raised against flag-tag in both plasmids. Nuclei were then stained using DAPI nuclear stain before mounting of coverslips onto microscope slides for confocal imaging and analysis. Analysis of the subcellular distribution patterns revealed that huIFIT5 expressed throughout the cells present in both the cell nucleus and cytoplasm (**Figure 3**). In contrast, paIFIT5 was exclusively observed in the cytoplasm. In order to exclude the possibility of flag-tag interference with the cellular distribution of paIFIT5, paIFIT5 was also fused with a V5 tag and cellular localisation was assessed. Parallel to flag-tagged paIFIT5 expression, V5-tagged paIFIT5 also expressed exclusively in the cell cytoplasm (**Figure 3**).

### 3.3 paIFIT5 is interferon and virus-inducible

**Figure 4:**
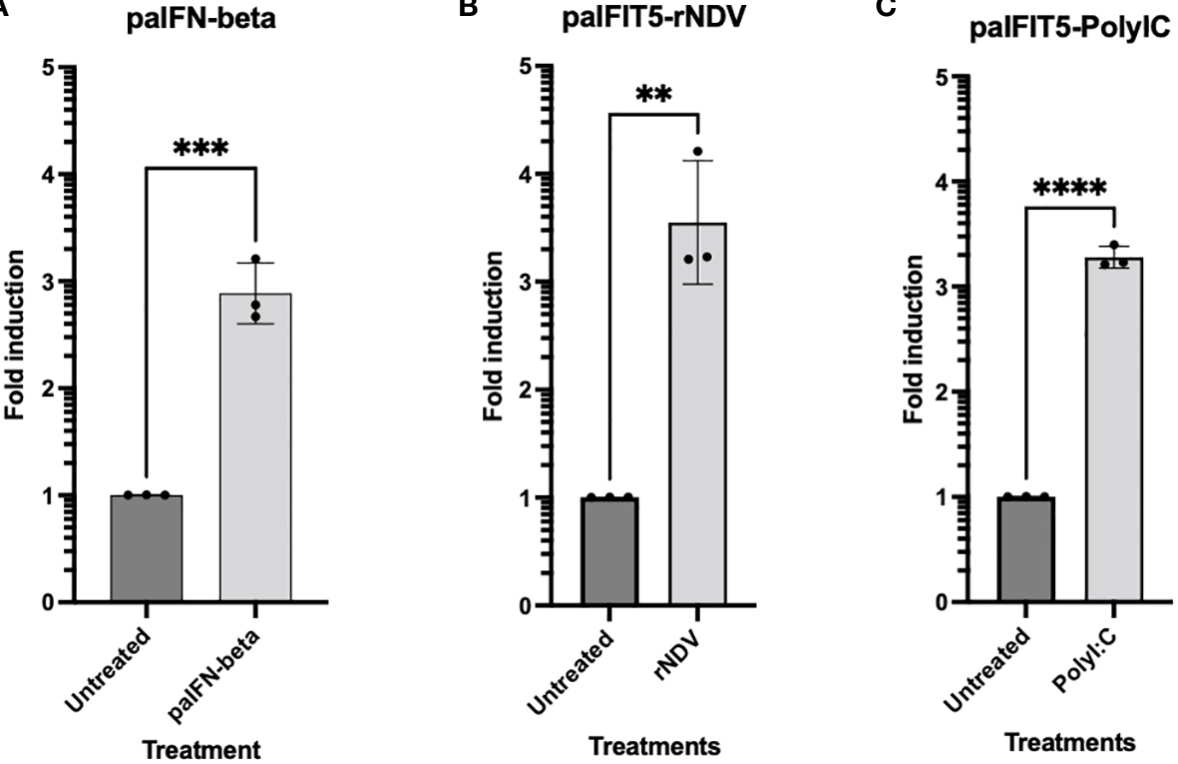
RT-qPCR Expression of paIFIT5. Quantitation of paIFIT5 mRNA in PaBr cells stimulated with either; **(A)** 200 units of *P.alecto* IFNβ (paIFNβ) **(B)**, 1.0 MOI of NDV or **(C)** 150 µg poly I:C for 24 hours before RNA extraction and analysis for RT-qPCR using primers specific for the *P.alecto* IFIT5 gene. Significance was determined at p ≤0.01 (**), p ≤0.001 (***) and p ≤0.0001 (****). Graph was generated by GraphPad Prism 8 software.

Previous studies have suggested that the potential antiviral activity of huIFIT5 against viruses is attributed to the viral 5’ppp molecular signature (9, 15). Nevertheless, prior to delving into the assessment of any potential antiviral effects of paIFIT5, it was imperative to ascertain whether paIFIT5 exhibited transcriptional activation in response to viral infection and/or interferon stimulation. Previously, huIFIT5, has been reported as both interferon and virus-inducible (14). Therefore, we aimed to investigate the transcriptional activation of paIFIT5 under the stimulation of interferon and viral infection. IFNβ (**Figure 4A**) was chosen as it is a type I interferon (IFN) which directly induces the transcription of IFIT5, whereas Newcastle disease virus (NDV) (**Figure 4B**) and poly I:C (**Figure 4C**) are stimuli to induce the transcription of IFIT5 indirectly via IFN expression and antiviral signalling.

Our results demonstrated the successful induction of paIFIT5 in the presence of all three stimulants compared to the untreated controls. However, amongst all, the highest induction for paIFIT5 (3.5-fold) was observed following NDV infection (**Figure 4B**), whereby activation of paIFIT5 is likely through the indirect activation of IFNs. paIFNβ also successfully induced the transcription of paIFIT5 almost 3-fold (**Figure 4A**). It is understood that negative-sense RNA viruses, including NDV, produce dsRNA intermediates during their replication cycle, which in turn, could additionally mediate the transcription of paIFIT5. In addition, poly I:C also activates paIFIT5 production over 3-fold (**Figure 4C**) similarly to NDV also as a result of IFN production leading to the transcriptional activation of paIFIT5. Poly I:C is a synthetic analogue of dsRNA, which is used as a surrogate to mimic viral infection and antiviral responses in cells through the induction of interferons. Poly I:C is first recognised by Toll-like receptor 3 (TLR3), present within endosomes of cells, activates interferon regulatory factor 3 (IRF3) and leads to the production of IFNs. Collectively, these results confirm that paIFIT5 is both interferon and virus inducible.

### 3.4 paIFIT5 exerts potent antiviral effects *in vitro*

**Figure 5:**
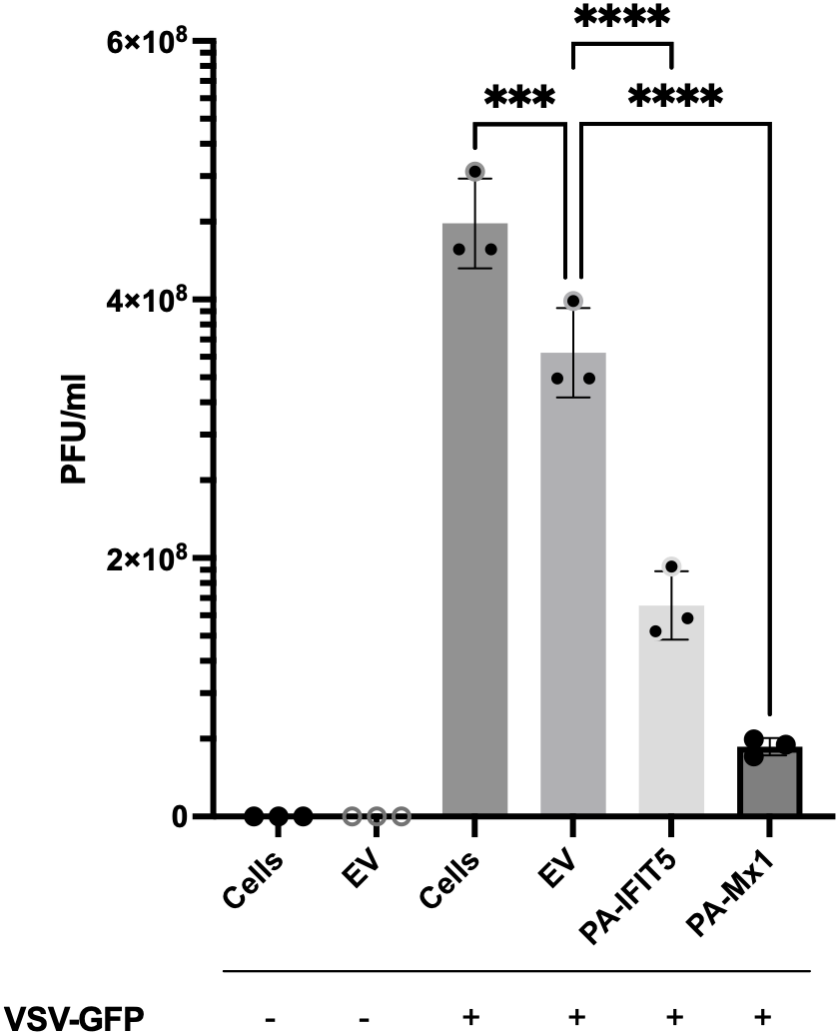
The antiviral activity of paIFIT5 measured against VSV-GFP replication. VeroE6 cells were transfected with 2µg of paIFIT5, paMx1, empty vector or left non-transfected for 24 hours. Cells were then infected with VSV-GFP at an MOI of 0.25 or left uninfected for 24 hours before quantification via plaque assay analysis in VeroE6 12-well plates. Significance was determined at p ≤0.001 (***) and p ≤0.0001 (****). Figure was generated using GraphPad Prism 8 software and significance was calculated using a One-Way ANOVA.

To further investigate the potential antiviral ability of paIFIT5 against viruses, paIFIT5 was overexpressed in VeroE6 cells and subsequently infected with vesicular stomatitis virus expressing GFP (VSV-GFP). Viral supernatants taken from these cells were collected and used for plaque assay quantification of viral titre (**Figure 5**). VSV is a negative-sense RNA virus that bears the 5’ppp molecular signature that IFIT5 is understood to recognise (9). Furthermore, VSV is a well-characterized model for plaque assay analysis in VeroE6 cells and can produce countable, quantifiable plaques (38). Briefly, VeroE6 cells were transfected with either paIFIT5, paMx1 or with an empty vector (EV) transfection control, for 24 hours before infection with VSV-GFP at an MOI of 0.25 or left uninfected. We used paMx1 as a positive control here, because Mx1 is a well-characterised interferon-stimulated gene (ISG) known for its antiviral activity (31). After 24 hours, viral supernatant was collected and used for plaque quantification.

Our results indicate that paIFIT5 displays a significant antiviral activity against VSV-GFP, compared with the infected cells (infection control) or EV infected control (transfection control). Cells infected with VSV-GFP displayed a viral titre around 5x10^8^ PFU/ml, whereas overexpression of paIFIT5 resulted in a much lower titre (less than 2x10^8^ PFU/ml), suggesting that to a certain degree, paIFIT5 inhibits the replication of VSV-GFP. Accordingly, additional investigations to elucidate the mechanistic processes through which paIFIT5 hinders viral replication would prove insightful. Whilst paIFIT5’s antiviral role is acknowledged, it remains to be clarified whether this effect arises from its interaction with 5’ppp on negative-sense RNA viruses exclusively, or if it exhibits a more comprehensive antiviral impact against other types of viruses.

### 3.5 paIFIT5 specifically inhibits negative-sense RNA viruses

**Figure 6:**
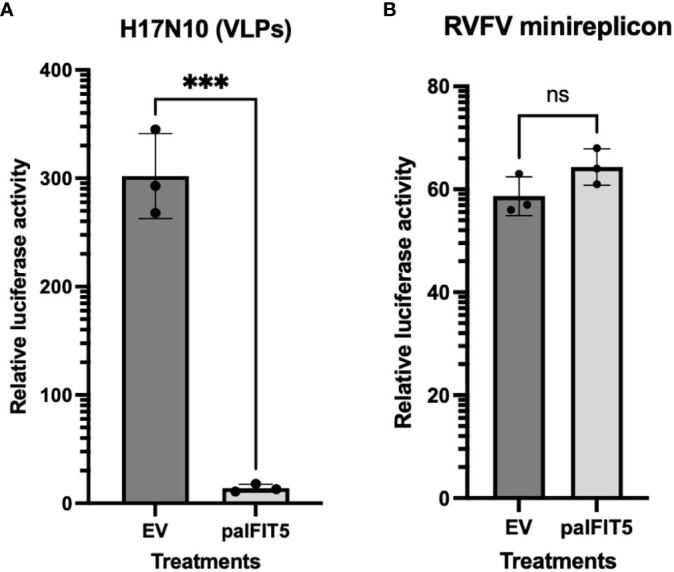
Influence of paIFIT5 on the polymerase activity of bat influenza (H17N10) and Rift-valley Fever Virus (RFV). **(A)** FLUAV (H17N10) VLP minireplicon system consisting of 10ng of PB2, 10ng of PB1, 10ng of PA, 10ng of NP, and 50ng of Pol-I FF-Luc was co-transfected alongside helper plasmids encoding the additional structural proteins from the H7N7 virus, in addition to 300ng of paIFIT5 expression plasmids, or empty vector (EV) control plasmid. At 48h hours post-transfection, supernatants were collected, and the cell lysates were analysed for firefly luciferase activity. The empty vector control was set to 100% and significance was calculated using a one-sided student t test (*n*=3). **(B)** HEK293T cells were transfected with 250ng of paIFIT5 alongside plasmids encoding RVFV N, L and M proteins and the Renilla luciferase-encoding minigenome (250ng each). At 48 hours post transfection cells were lysed and used to measure the Renilla luciferase activity. As a control, the paIFIT5 plasmid was replaced with an EV. The activity of the empty vector control was set to 100% and statistical significance was calculated using a one-sided student t test (n=3). NS stands for non-significant.

After the previous observation of the antiviral activity of paIFIT5 against VSV(-GFP), we next aimed to investigate if paIFIT5 exhibited an antiviral activity against endogenous bat viruses. Bats have been shown to host several viral pathogens, including influenza A viruses such as the H17N10 subtype in central American fruit bats (23). Influenza A viruses are negative-sense, single stranded viruses, which possess the 5’ppp signature (39). Hence, we selected H17N10 bat influenza virus to investigate potential antiviral activity of paIFIT5 mediated via the interaction of paIFIT5 with 5’ppp. However, attempts to isolate the H17N10 virus have so far remained unsuccessful (40, 41). Therefore, in order to mimic the action of H17N10, we used artificial replication-deficient, transcriptionally active virus-like particles (VLPs) to observe the antiviral effect of paIFIT5 against bat influenza virus, as previously described (31). The VLPs carry a firefly luciferase minigenome, the polymerase subunits and NP of bat H17N10 virus, in addition to all other structural proteins of the H7N7 influenza virus. HEK293T cells were used to produce the H17N10 VLPs, and co-transfected with paIFIT5. At 48 hours post-transfection, supernatants were collected, and the cell lysates were used to measure firefly luciferase activity, which was defined as a representative measure of the viral polymerase activity of H17N10 (**Figure 6A**). Analysis of the H17N10 VLP assay revealed that paIFIT5 significantly reduced reporter gene expression, compared to the empty vector control. The reduction in luciferase activity in the presence of paIFIT5 indicates a strong antiviral ability of the paIFIT5 protein against H17N10.

Previous studies have reported that bats are able to host a broad range of emerging pathogens, including bunyaviruses, which are negative-sense RNA viruses known for causing severe disease in humans and animals. Bunyaviruses are often transmitted by arthropod, however, studies have also shown bunyaviruses such as Hantavirus, Nairovirus and Phenuivirus can be harbored by small mammalian hosts including rodents and bats (42–46). Therefore, we aimed to assess the potential antiviral activity of paIFIT5 against bunyaviruses (47). To investigate this, a minireplicon system for RVFV was utilised as previously described (31, 48). HEK293T cells were co-transfected with the paIFIT5 encoding plasmid, alongside expression constructs coding for the viral L, M and N proteins of RVFV and a *Renilla* luciferase-encoding minigenome. For the control, IFIT5 was omitted and replaced with an empty vector (EV). Our results showed that in the presence of paIFIT5, the viral polymerase activity of RVFV appeared very slightly increased compared to the EV control, however statistical analyses deemed these results non-significant, likely due to the variation observed in the control (**Figure 6B**).

### 3.6 paIFIT5 interacts specifically with 5’ppp-bearing RNA

**Figure 7:**
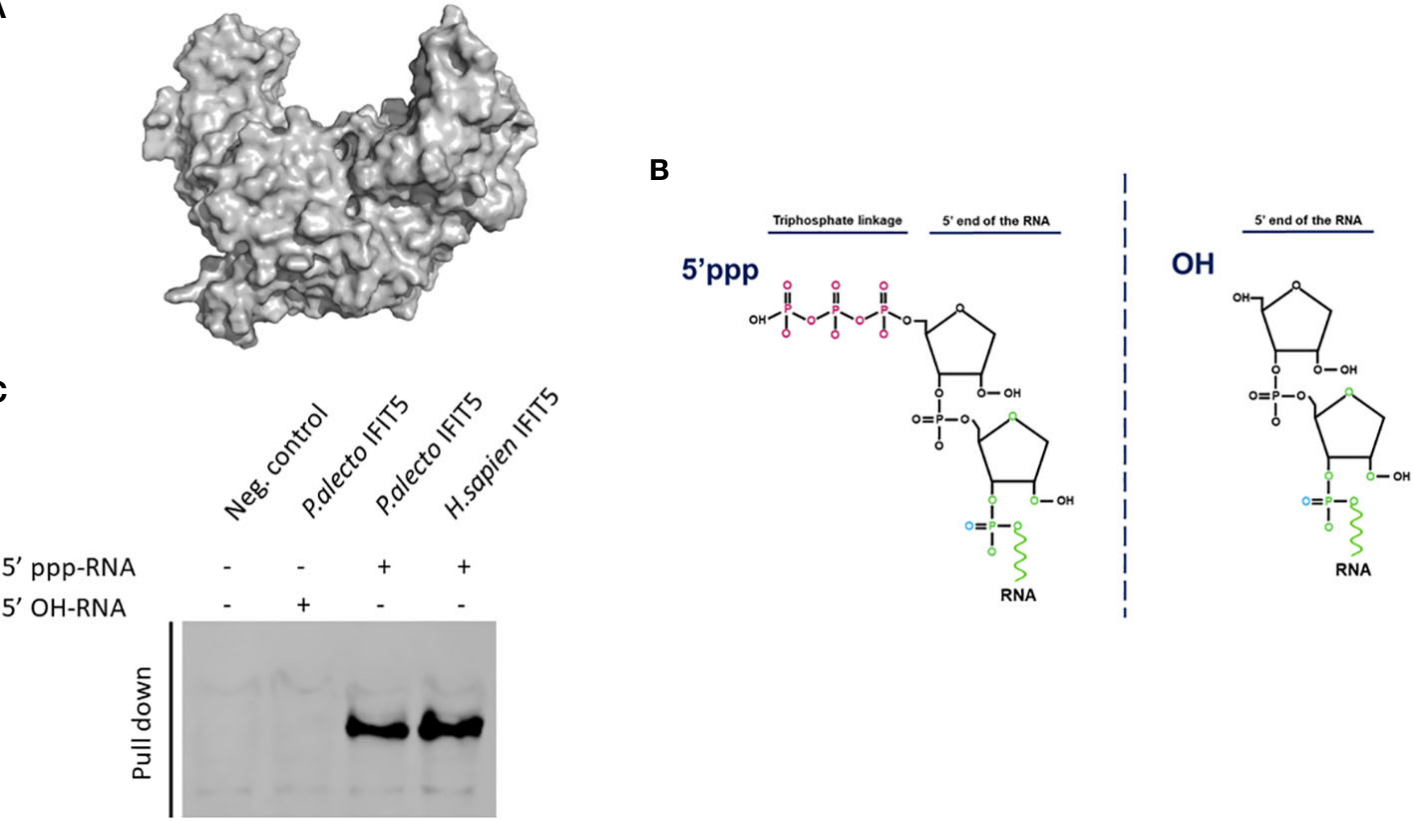
Interaction of paIFIT5 with RNA carrying modifications in their 5’ termini using RNA-protein immunoprecipitation. **(A)** The 3D structure of paIFIT5. **(B)** The genomes of negative-sense RNA carry a triphosphate linkage (5’ppp) in the first transcribed base of the RNA. **(C)** Pull-down of biotinylated RNA interacting with paIFIT5 indicated that both huIFIT5 and paIFIT5 interacted with RNA carrying 5’ppp structures.

Human IFIT proteins are known to interact with RNA carrying modifications at their 5’ ends and notably huIFIT5 has been shown to interact with a 5’ppp molecular signature (7, 9). Therefore, we aimed to explore if paIFIT5 can interact with 5’ppp-bearing RNA in the same manner. As aforementioned, paIFIT5 appears highly conserved in its structure similar to human counterpart. The huIFIT5 displays a novel arrangement of TPR domains that bind specifically to ppp-RNA in a non-sequence-specific manner Abbas et al. (9). We generated a 3D crystallised structure of the full-length paIFIT5 (**Figure 7A**) was highly conserved in structure with the huIFIT5 (9) and appears to possess a similar pocket, which may be involved in binding with 5’ppp.

To determine the molecular mechanisms involved in the recognition of 5’ppp by paIFIT5, we generated RNA species bearing either the 5’ triphosphate (5’ppp-RNA) group or alternatively a hydroxyl (5’OH-RNA) group at their N-termini (**Figure 7B**). These RNAs were biotinylated and coupled with agarose beads, which were subsequently incubated with HEK293T cells expressing V5-tagged paIIFT5 or huIFIT5. Then, the ribonucleoproteins were purified and the interaction of paIFIT5 or huIFIT5 was determined by staining for IFIT5. Our results showed that paIFIT5 did not interact with the 5’OH-RNA species, while both paIFIT5 and huIFIT5 recognised RNA carrying the 5’ppp signature (**Figure 7C**).

## 4 Discussion

ISGs provide an essential role in the innate immune response, acting in an antiviral manner by targeting different stages of viral replication (6, 49). IFIT proteins are a family of ISGs, which are major players in innate immunity, due to their substantial antiviral responses against viral and IFN stimuli (3, 7). Significant advances have been made in the knowledge and characterisation of IFIT proteins in human and mouse, however understanding of IFIT proteins in other mammalian species as bats currently remains understudied (7, 50). Characterisation of bats IFIT proteins is crucial to gain a better understanding of their innate immune responses, which have previously been highlighted as somewhat unique in aiding bats to host viruses asymptomatically (51). Unearthing the role of ISGs such as IFIT proteins in the bat antiviral immune response, would help for a better understanding of their roles as viral reservoirs and potentially provide a basis to regulate the common emergence and spillover of zoonotic pathogens from bats. Generally, the functions of IFIT1/IFIT2/IFIT3 have been extensively investigated in comparison to IFIT5 (19). Significantly, it has been reported that human IFIT5 demonstrates antiviral effects similar to IFIT1, which involves the direct recognition and binding of RNA with a distinct 5’ppp molecular marker (9, 14, 19). Due to this discovery, IFIT5 was chosen as an initial gene of interest meriting further exploration in bats. This exploration aims to ascertain whether bat IFIT5 also exhibits the ability to recognise 5’ppp RNA and subsequently exhibit antiviral activity. IFIT5 gene of the Australian Black flying fox (*P.alecto*) was chosen for analysis as it remains one of the few whole-bat genomes that have been sequenced and annotated (52). Furthermore, *P.alecto* are the natural reservoir for several pathogenic viruses, including henipaviruses, deeming them an appropriate representative species that warrants investigation (53–55). In order to explore bat IFIT5, we first employed a bioinformatic approach to compare the *P.alecto* IFIT5 gene (paIFIT5) with other animals, assessing their gene synteny, sequence conservation and phylogenetic relationship. Based on these results, paIFIT5 is highly conserved with IFIT5 orthologues in other mammals. Interestingly paIFIT5 displayed a closer phylogenetic relation and pairwise sequence of 74% with horse IFIT5. This supports the notion that alternative to bats previously belonging to, and diverging from, the same group as primates. Bats may actually belong to the super-order named Pegasoferae, which also contains horses (35, 56–58). However, due to the large species diversity of bats, these results are likely not representative for IFIT5 present in other bat species, but are useful in our initial understanding of IFIT5 in *P.alecto*.

In this study, we compared the sequence and TPRs of paIFIT5 with huIFIT5, due to the recent findings of the antiviral role of huIFIT5, in addition to their observed sequence homology (19). Our analyses based on the protein conservation and 3D structure demonstrate the high conservation in the protein sequence between paIFIT5 and huIFIT5, differing by only a few amino acids. Interestingly, the IFIT5 proteins differ slightly in their TPR repeats, due to the presence of an additional TPR (labelled TPR3b) in paIFIT5. TPRs are characteristic for IFIT proteins and have key structural roles that are responsible for the binding with a diverse range of ligands, such as protein and peptide recognition (3, 9). Study by Abbas et al. (9) has illustrated the crystal structure of huIFIT5 and a novel arrangement of TPR domains that are able to bind with the 5’ppp-RNA in a non-sequence-specific manner. Conservation of TPR analogues between paIFIT5 and huIFIT5 may suggest a sustained ability of paIFIT5 to bind with similar ligands, including the 5’ppp-RNA. However, the extra TPR (TPR3b) in paIFIT5 potentially requires further investigations to determine the crystallised conformational structure of paIFIT5 and whether this additional TPR of paIFIT5 can influence on the binding ability or ligand spectrum. Furthermore, deletion and or mutational investigations for TPRs of paIFIT5 may be warranted in order to gain a valuable insight into the binding mechanism of paIFIT5 and the implications on TPR positioning.

Additionally, we aimed to investigate the cellular localisation of paIFIT5 when overexpressed in VeroE6 cells, as this was yet to be explored. Moreover, there also remains limited information available on the cellular localisation of huIFIT5, therefore we investigated both IFIT5 plasmids in this study. Upon immunofluorescence staining and imaging of paIFIT5 and huIFIT5 overexpression, we observed a largely cytoplasmic expression of paIFIT5, in contrast to huIFIT5 which was evident in both the cell cytoplasm and nucleus. Neither paIFIT5 or huIFIT5 appeared to localise to specific subcellular locations within these regions but could be further explored to determine if this is true. Future studies into the potential colocalisation of IFIT5 proteins with subcellular organelles and potentially the plasma membrane, may enable a better perception of paIFIT5 and huIFIT5 localisation and activity.

Owing to facilitate the IFN induction or viral infection; IFNs, dsRNA and lipopolysaccharides are all characterised stimuli known to induce the transcription of human IFIT proteins (59, 60). The induction of IFIT1/IFIT2/IFIT3 by these stimuli are well-represented in the literature, whereby they are described to form multimeric complexes that infer antiviral activities (61). However, the triggering of IFIT5’s expression remained unclear, and its roles and functions demonstrated inconsistencies and contradictions (62–64). Zhang, Liu et al. (19) were able to confirm that IFIT5 was significantly induced upon virus infection, poly (I:C) and IFN-stimulation at both the protein and mRNA levels. Therefore, we aimed to investigate whether bat (paIFIT5) can be induced by these stimuli. Our results confirmed that paIFIT5 was transcriptionally activated in response to NDV, poly(I:C) and paIFNβ, whereby the fold induction of paIFIT5 was consistently increased (at least two-fold), compared to untreated controls. These results confirm that IFIT5 in *P.alecto* is both virus and IFN-inducible, however it might not be the case for all bat species and hence more investigations are required to determine if this induction is unique to *P.alecto* or is constant for all bat species. In addition, NDV appeared to increase the activation of paIFIT5 the highest, followed by poly(I:C) and then IFNβ. Higher induction of paIFIT5 by NDV is likely attributed to multiple factors including viral dsRNA generated during replication, sensing of viral nucleic acid by intrinsic sensors and cellular responses to viral infection. Similarly, poly (I:C) (synthetic dsRNA) can be sensed in the same manner as virus-derived dsRNA, leading to profound induction of paIFIT5. In contrast, treatment with IFNβ exclusively induces the paIFIT5 through the JAK-STAT signalling pathway. For a broader analysis, a variety of viral stimuli should be assessed in addition to other types of IFNs, including type I IFNs and type III IFNs, which are known to induce ISGs (65, 66) but were not investigated here.

Previous research highlighted the antiviral effect of huIFIT5 against negative-sense RNA viruses, therefore we aimed to demonstrate if paIFIT5 also possesses any antiviral activity towards this group of viruses, using NDV (19) and to quantify the viral replication using plaque assay analysis. Because NDV does not form clear plaques, we used VSV, also a negative-sense RNA virus possessing the 5’ppp-RNA structure. Our results showed that paIFIT5 overexpression significantly inhibited VSV-GFP virus replication, resulting in a lower viral titer compared to paMx1 (positive control), the virus infection control (cells only) and transfection control (EV). Mx1 is one of the most studied bats ISGs (31, 67, 68) and has antiviral activities specifically for inhibiting negative sense RNA viruses.

In order to further elucidate the antiviral nature of paIFIT5, the influenza A-like bat virus (H17N10) and RVFV were selected as model viruses possessing 5’ppp structures for this investigation. Due to the high containment required for RVFV and the inaccessibility to H17N10, we could not measure the influence of paIFIT5 on the replication of these viruses via plaque assay. Therefore, minigenome assays were alternatively employed to allow for the investigation of viral inhibition in HEK293T cells overexpressing paIFIT5. Our results showed that paIFIT5 significantly (p <0.001) inhibited the H17N10 viral minigenome and hence the formation of new VLPs, as defined by luciferase activity reduction. These results demonstrating the suppression of H17N10 viral replication in bats by paIFIT5, correspond with the understanding that bats are able to maintain viral replication at manageable levels (21). On the other hand, the RVFV minireplicon assay gave non-significant results and therefore the influence of paIFIT5 on RVFV could not be determined.

Despite lack of reporting on IFIT5 functionality, huIFIT5 is known to recognise a range of RNA constructs, including 5’ monophosphate (5’p), double stranded DNA and RNA with CAP0 modifications and 5’triphosphates (5’ppp) (9, 69, 70). By generating modified RNA constructs bearing either a 5’ppp signature or alternatively a hydroxyl (OH) group, as previously described (18, 28), we aimed to determine if paIFIT5 specifically interacts with the 5’ppp signature commonly found within viruses possessing negative-sense single stranded RNA genomes. RNA-protein interaction results showed that paIFIT5 specifically interacted with RNA possessing the 5’ppp signature and did not interact with OH-bearing RNA. huIFIT5, already known to interact with 5’ppp-RNA, was used as a positive control for comparison purposes. From these results, it is feasible that paIFIT5 is able to sense foreign RNA, bearing 5’ppp, a molecular signature present only within genomes of negative-sense single stranded RNA viruses and produced as an intermediate during positive-sense RNA genome replication (71). By sensing this RNA signature, paIFIT5 may be able to distinguish this from self-RNA, which instead bear monophosphate or CAP0 structures at their 5’ end. The direct interaction of paIFIT5 with 5’ppp RNA, could potentially sequester the viral RNA, thus inhibiting its replication and translation by the host machinery.

Our results demonstrating the interaction of paIFIT5 with 5’ppp-RNA and its antiviral activity are useful in understanding the role of bat ISGs in bat innate immunity. However, these findings warrant further experimental validation via the use of paIFIT5 knockout experiments in bat cells, in order to clarify the antiviral potential of endogenous IFIT5 in host cells. Furthermore, research investigating any potential influence of other bat IFIT proteins are required to determine if paIFIT5 acts as a monomer in recognising and binding with 5’ppp-bearing RNA viruses, or alternatively paIFIT5 requires assistance via the formation of multimers with other IFITs. Lastly, significant progress can be made in understanding the downstream activation of immune mechanisms caused by the recognition of 5’ppp RNA by paIFIT5 and the potential antiviral resistance this may confer to the bat hosts. Overall, our research employed both functional genomics and molecular biology to provide solid foundations for the characterisation of IFIT5 in the megabat species *P.alecto*, which previously remained unexplored. Results additionally investigated the functional rationale for the antiviral capacity of paIFIT5 against viruses possessing 5’ppp RNA molecular signatures. The groundwork presented in this study justifies future investigations assessing not only the antiviral potential of paIFIT5 against a broader range of viruses (RNA and DNA), but also in other bat species and the effects that these interactions impose on bat innate immunity.

## Data availability statement

The original contributions presented in the study are included in the article/supplementary material. Further inquiries can be directed to the corresponding author.

## Ethics statement

Ethical approval was not required for the studies on animals in accordance with the local legislation and institutional requirements because only commercially available established cell lines were used.

## Author contributions

EC: Data curation, Formal analysis, Methodology, Writing – original draft. MA: Data curation, Formal analysis, Methodology, Writing – review & editing. RE: Methodology, Writing – review & editing. AF: Data curation, Methodology, Writing – review & editing. MR: Data curation, Methodology, Writing – review & editing. MM: Conceptualization, Formal analysis, Funding acquisition, Investigation, Project administration, Supervision, Writing – review & editing.
